# Non-reciprocity and topology in optics: one-way road for light via surface magnon polariton

**DOI:** 10.1088/1468-6996/16/1/014401

**Published:** 2015-01-13

**Authors:** Tetsuyuki Ochiai

**Affiliations:** Photonic Materials Unit, National Institute for Materials Science (NIMS), Tsukuba 305-0044, Japan

**Keywords:** non-reciprocity, one-way, photonic crystal, ferrite, surface magnon polariton

## Abstract

We show how non-reciprocity and topology are used to construct an optical one-way waveguide in the Voigt geometry. First, we present a traditional approach of the one-way waveguide of light using surface polaritons under a static magnetic field. Second, we explain a recent discovery of a topological approach using photonic crystals with the magneto-optical coupling. Third, we present a combination of the two approaches, toward a broadband one-way waveguide in the microwave range.

## 1. Introduction

Optical components that exclude undesirable light are indispensable in optical communication technology. Optical isolators, gyrators and circulators are among such components [1]. They commonly break reciprocal light transport. The violation of the reciprocity, that is, light transport in one direction behaves differently from that in the opposite direction, is crucial. This non-reciprocity is closely related to time-reversal symmetry (TRS) and is controlled by a static magnetic field.

In fact, conventional non-reciprocal components use the Faraday effect in which light polarization rotates along its trajectory owing to the magneto-optical coupling. Here, either a bias magnetic field or a spontaneous magnetization exists along the trajectory. To obtain a sufficient rotation angle for an isolator to work, for instance, a long optical path is necessary. This is because the rotation angle is proportional to the path length. This fact results in the rather large size of nonreciprocal optical devices.

To overcome the size demerit, the optical non-reciprocity in the Voigt geometry is desirable. In the Voigt geometry, light propagates in a direction perpendicular to the magnetic field. It enables us to construct compact non-reciprocal components. Furthermore, if the non-reciprocity works in a certain band width, we do not need to fine-tune system parameters, often employed in conventional designs using wave-interference effects.

An ideal situation is such that in a certain band width a light propagation channel exists in one direction, but does not in the opposite direction. Without any dissipation and interference, such a mode results in a non-reciprocal and one-way waveguide in a finite frequency range. In addition, the absence of the counter-propagating mode prohibits the back scattering by structural defects. Thus, the light propagation is robust against disorder.

The design of such a one-way waveguide has a long history [2]. Until recently, however, very limited systems are known: surface plasmon polaritons of metal under a magnetic field, and surface magnon polaritons of magnetic material. The polaritonic wave of the edge magnetoplasmon in a two-dimensional electron system [3] should have also a finite-band non-reciprocity below the cyclotron frequency.

In the last decade, a completely different approach to the finite-band one-way waveguide has been proposed theoretically [4–7]. Some of them are also demonstrated experimentally in the microwave range [8, 9]. This new approach utilizes a topological nature of the radiation field in photonic crystals, namely, artificial periodic structures made of different optical substances. With this approach, operating frequencies and their band width are controllable via the photonic-band engineering of underlying photonic crystals. Since there are many degrees of freedom in photonic crystals, e.g., optical substances, their geometry, and lattice constants, operating frequencies and their band width are not limited as in surface polaritons. A minimum requirement is a non-zero magneto-optical coupling (imaginary off-diagonal components of the permittivity or permeability). The resulting band width is roughly proportional to the magneto-optical coupling. Therefore, a challenge in material science is to make optical substances with large magneto-optical couplings. Although a large magneto-optical coupling of the same order as the diagonal components is available for permeability in the microwave range using ferromagnetic resonance, it is still challenging to realize a large coupling above this frequency range for permittivity or permeability.

In this paper, we propose a combination of these two approaches toward a broad-band one-way light waveguide using modulated surface magnon polaritons. A semi-infinite ferrite material with a periodic hole array in the vicinity of the sample edge strongly modulates the surface magnon polariton without the hole array. In addition, photonic bands with multiple band gaps are formed in the hole array. As a result, in a vast frequency range, a one-way light waveguide can be realized, though the frequency range is multiply divided by the photonic bands.

The paper is organized as follows. In sections 2 and 3, we briefly summarize the traditional approach using surface polaritons and the topological approach using photonic crystals, respectively. In section 4, a combined approach of these two is presented. Finally in section 5, we summarize the results obtained in this paper.

## 2. Non-reciprocity in surface polaritons

In optics non-reciprocity, in a narrow sense, is defined by the frequency dispersion of eigenmodes that satisfies 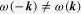. In free space, the photon dispersion is given by 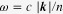 with refractive index *n*. Obviously, it is reciprocal.

To realize the non-reciprocity, we need to break both the TRS and the space-inversion symmetry (SIS: 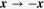). Either the TRS or SIS alone results in 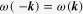. The SIS is easily broken by geometry. For instance, a flat interface between two different optical substances breaks the SIS. The TRS is broken typically by applying a static magnetic field, because the magnetic field is odd under the time reversal. However, a non-zero magnetic field does not always induce detectable non-reciprocity. In optics, effects of the TRS breaking emerge through imaginary off-diagonal components in permittivity or permeability tensors. By the Onsager relation, the permittivity satisfies1
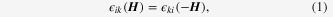
where 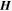 stands for a static magnetic field of either applied one 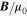 or spontaneous magnetization [10]. The same equation is satisfied for permeability. This equation along with the hermicity of the permittivity in dissipation-less systems implies that the imaginary off-diagonal components are odd functions of 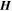. Typically, the permittivity is written as2
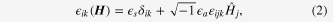
where 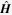 is the unit vector orienting 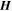. The ratio 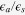 is less than 10^−3^ for most transparent materials [11]. Therefore, if the imaginary off-diagonal components, 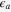 in this case, are very small, effects of the TRS breaking are very limited. Thus, a challenge in material science is to make compound materials with large magneto-optical couplings.

**Figure 1.**
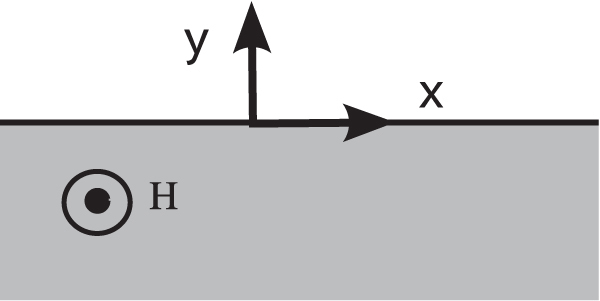
The geometry under consideration. The magnetic field is applied in the lower part.

In this section, we consider a flat interface between magnetic and non-magnetic media shown in figure 1 as a system without the TRS and SIS. For simplicity, we consider light propagation in the *xy* plane, and a static magnetic field is applied in the *z* direction. Obviously, the parity symmetry 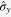 (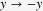) is broken. The parity symmetry 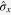 is also broken by the magnetic field. The parity symmetry 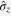 is preserved irrespective of the magnetic field. As a result, the SIS 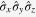 is broken.

To realize the one-way road for light, we utilize a surface mode localized near the interface. Usually, such modes exist if one of the two media screens the radiation field and the other does not. Therefore, if one of the two media is taken to be a normal dielectric, the other must be a screened medium. Here, we consider two cases, screened by negative permittivity (metal) and negative permeability (ferrite).

### 2.1. Surface plasmon polaritons under magnetic field

One example of a finite-band non-reciprocal light waveguide is found in the surface plasmon polariton under a magnetic field. In a free-electron metal under a magnetic field along the *z* direction, the permittivity tensor becomes [12]3
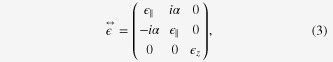

4
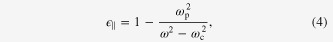

5
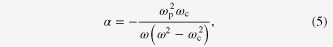

6
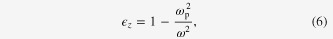
where, 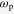 and 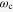 are the plasma and cyclotron frequencies, respectively. Dissipation is neglected for simplicity.

We should point out that the plasma frequency is generally in the visible-to-ultraviolet range, whereas the cyclotron frequency is in the microwave range depending on applied magnetic field. Therefore, a scale difference of order 10^6^ exists between 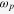 and 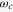. This difference gives very small magneto-optical coupling 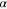 in comparison to the diagonal permittivity 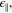 and results in a narrow band width of the one-way light waveguide.

Let us consider the surface plasmon polariton localized around the flat interface between metal and dielectric. This geometry supports a localized mode of the radiation field near the surface, irrespective of the applied magnetic field. That is, the surface plasmon polariton. The dispersion relation of the surface plasmon polariton under the magnetic field is given by7
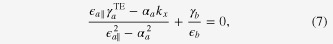

8
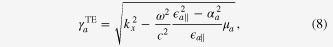

**Figure 2.**
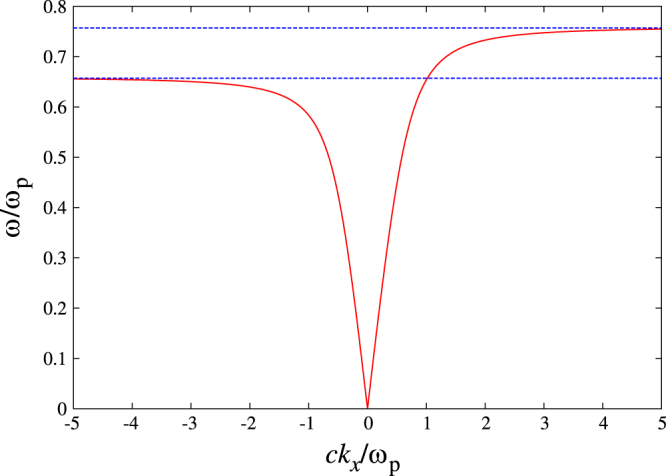
The dispersion relation of the surface plasmon polariton under a static magnetic field along the *z* direction. Just to visualize the one-way band, whose upper and lower frequencies are indicated by the dotted lines (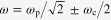), we assume 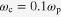. Actually, 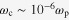, so that the one-way band width is almost invisible, if plotted in actual scale.

9
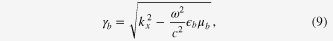
where we assume the metal has the off-diagonal permittivity of equation (3) with 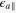 and 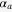, and the diagonal permeability 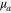. The dielectric has diagonal permittivity 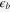 and permeability 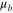. The dynamical magnetic field of the surface plasmon polariton is polarized in the *z* direction. Figure 2 shows the dispersion relation of the surface plasmon polariton under a static magnetic field.

Obviously, the dispersion is non-reciprocal, and has the one-way band in the narrow frequency interval of 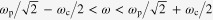. The band width is just 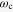, while its mid-band frequency is 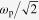, namely, the surface plasmon frequency in the non-retardation limit. Therefore, the one-way band in the surface plasmon polariton is very limited.

### 2.2. Surface magnon polariton

As is well known, permeability in the visible and higher frequency range is almost equal to one. Dynamical magnetization can not follow applied AC magnetic field. If we consider much lower frequencies (the microwave range), however, we can enjoy large diagonal permeability along with the off-diagonal permeability (the magneto-optical coupling) near the ferromagnetic resonance in ferrite materials. There, a magnetic counterpart of the surface plasmon polariton, namely, the surface magnon polariton, emerges. In a ferrite material, the spin wave is the dominating low-energy excitation. Its long-wavelength approximation is the magneto-static wave. The surface magnon is a localized version near the surface of the ferrite material. It can couple with light, forming the surface magnon polariton.

In the free-spin model of ferrite materials, the permeability is given by [1]10
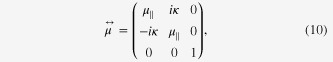

11
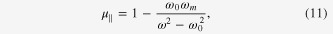

12
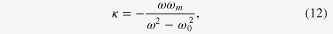
where 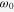 is the spin precession frequency and 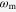 is the saturation magnetization frequency. The precession and magnetization frequencies are given by 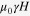 and 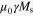, respectively, where 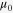 is the vacuum permeability, *γ* is the gyromagnetic ratio, *H* is the applied magnetic field in the *z* direction, and 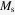 is the saturation magnetization in the same direction. The model has the polaritonic gap in 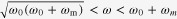, where the effective permeability 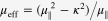 is negative and thus light propagation is not allowed.

The dispersion relation of the surface magnon polariton is obtained by solving the secular equation13
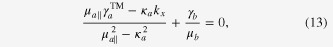

**Figure 3.**
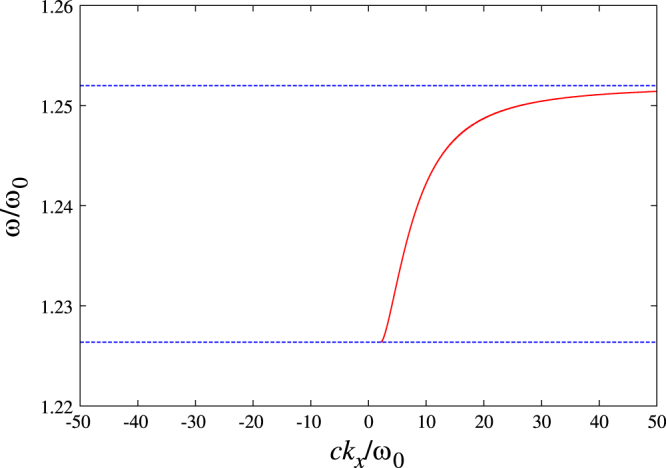
The dispersion relation of the surface magnon polariton under a static magnetic field along the *z* direction. The lower and upper frequencies of the one-way band are indicated by the dotted lines (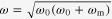 and 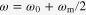, respectively). We assume 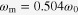.

14
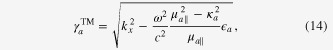
where we assume the ferrite has the non-diagonal permeability of equation (10) with 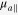 and 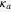, and the diagonal permittivity 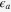. The dynamical electric field of the surface magnon polariton is polarized in the *z* direction. In the non-retardation limit 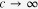, we obtain the surface magnon (magneto-static Damon–Eshbach wave [13]) whose frequency is 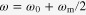 available only for 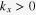. For 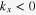 the surface magnon is not allowed to exist. The dispersion relation of the surface magnon polariton is shown in figure 3. The dispersion relation is clearly non-reciprocal and exhibits a one-way propagation in the frequency range 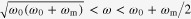.

A typical ferrite material is the yttrium iron garnet. It has the saturation magnetization of ∼1800 Gauss. At a bias magnetic field of 3570 Oersted (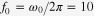 GHz), we have 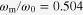. In this case the band-width/mid-band ratio of the surface magnon polariton is about 0.02, much larger than that in a one-way band of the surface plasmon polariton which is of order 10^−6^.

## 3. Topological construction via photonic crystal

In 2008, Haldane and Raghu proposed a completely different approach to a one-way light waveguide [4]. They focused on the so-called Dirac cone found in the photonic band structure of a triangular-lattice photonic crystal. If the gap is introduced in the Dirac cone by breaking the TRS, a finite-band one-way light waveguide can be realized near the edge of the photonic crystal. Its one-way band coincides with the band gap in the Dirac cone. This is an optical analogy of chiral edge states in quantum Hall systems [14].

In condensed matter physics, it is well-established that the emergence of the Dirac cone in the electronic band structure is a critical signature in topology [15]. It is sometimes the case that the two electronic bands touch conically at a certain *k* point (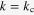) in the Brillouin zone at a certain parameter 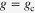, when we sweep a physical parameter *g*. Before (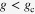) and after (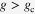) the touching, the momentum space topology, characterized by the so-called Chern number, changes abruptly [16]. If a non-zero Chern number emerges, chiral edge states are accompanied, according to the bulk-edge correspondence [17]. These chiral edge states are nothing but the one-way waveguide modes in optics. The number of the chiral edge states is given by the sum of the Chern numbers of the bands below the gap concerned.

In the proposal by Haldane and Raghu, the band touching and the separation are caused by the magneto-optical coupling. If the coupling is zero, the (gapless) Dirac cones are found at the corners of the first Brillouin zone of the triangular lattice. If it becomes nonzero, the gap is found in each Dirac cone, and the Chern number of the two bands becomes nonzero. Soon after this proposal, it is recognized that the scenario is not limited in the conical band touching (the Dirac cone), and that a quadratic band touching, which is usually found in the Brillouin zone center of the square and triangular lattices and in the Brillouin zone corner of the square lattice, is also a source of nontrivial topology [18]. In fact, chiral edge states emerge in the gap opened in the quadratic band touching, by breaking the TRS.

These theoretical proposals were confirmed experimentally in the microwave frequency range. For instance, in reference [8] the authors employed a square array of ferrite rods made of the vanadium-doped calciumirongarnet under a bias magnetic field of 0.20 Tesla. They observed a photonic band gap with the gap-width/mid-gap ratio of 6% around 4.5 GHz. In accordance with a theoretical simulation, they found unidirectional backscattering-immune edge states against metal obstacles, in the gap.

To elucidate the topological approach, let us consider photonic crystal made of ferrite rods arranged in the triangular lattice. We consider a frequency region in which the permeability tensor is not far from 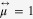, and the magneto-optical coupling can be viewed as a perturbation. In such frequencies, we can clearly see that the gap-opening in the Dirac cone is due to the magneto-optical coupling.

**Figure 4.**
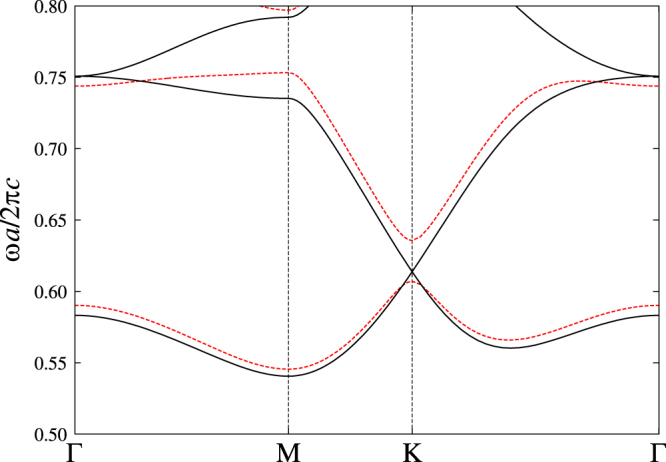
The photonic band structure of the transverse-magnetic polarization in the triangular array of circular ferrite rods with (solid curve) and without (dashed curve) the applied static magnetic field parallel to the rods. The background medium is air. The rod permittivity is taken to be 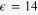, and the rod radius is 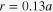, where *a* is the lattice constant. As for the rod permeability, we assume 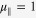 and 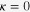 for the system without the magnetic field. For the system with the magnetic field, we assume dispersion-free values of 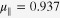, 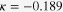 as an approximation. These values are taken from those of equations (11) and (12) at 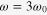, provided 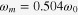.

Figure 4 shows the photonic band structure of the system before and after introducing the magneto-optical coupling. We can see the band gap opening of the Dirac cone, suggesting a nontrivial topology in the gapped system. In fact, the Chern numbers of the bands are evaluated as 0 (lowest, not shown), −1 (2nd), and 2 (3rd).

**Figure 5.**
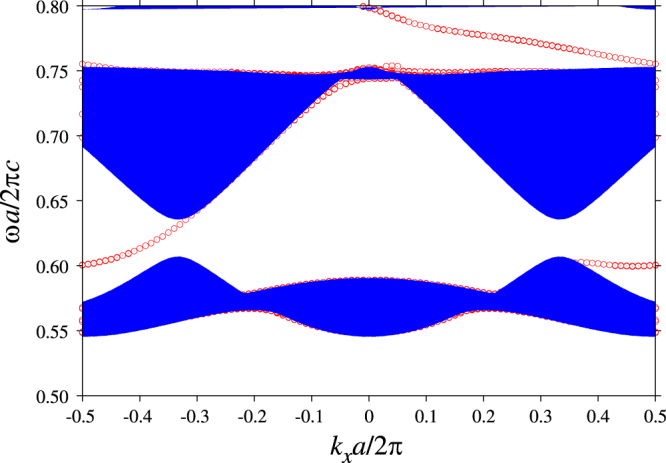
The dispersion relation of the edge states found in the photonic crystal edge normal to the *Γ*−M direction. The projected band diagram in the bulk is also plotted. The gapped photonic crystal with the same parameters as in figure 4 is employed. To clarify the edge states, we place a (perfect conductor) metal cladding away from the boundary layer with distance (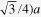.

Figure 5 shows the dispersion relation of the edge states in the gapped system. We can see that in the band gap around 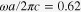, the dispersion curve of the edge states traverses the gap and connects the K and K’ valleys of the gaped Dirac cones. As a result, the one-way transmission band is formed in the gap. We should recall that the gap opening is due to the nonzero magneto-optical coupling *κ*. The gap width is proportional to *κ* in the degenerate perturbation around the Dirac point. The proportional constant reflects the properties of the degenerate mode at the Dirac point. Thus, the one-way band width there can be controlled by a photonic-band-mode design.

It is also remarkable that the topological approach can yield both right- and left-going one-way modes without inverting the magnetic field. For comparison, in the surface polaritons, the chirality is fixed depending on the sign of the magnetic field. In addition, more than one edge mode can emerge in a given gap. This is because the number of the edge states is equal to the sum of the Chern numbers of the bands below the gap, and the sum is not limited in ±1. Actually, we have the right-going mode in the Dirac cone gap around 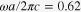. At the same time, the left-going mode is found in the gap around 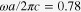, without inverting the magnetic field. The numbers and the chiralities of the edge states in the gaps are fully consistent with the Chern numbers of the photonic bands.

Finally, we should note that the topological approach to the one-way transport is quite universal. Basic ingredients are a periodic structure, a TRS breaking, and a band touching/separation. Therefore, this approach can be extended to various wave phenomena on periodic structures, for instance, spin wave in magnonic crystal [19]. There, chiral edge modes of spin waves are predicted. We also note that, recently, the topological approach is extended to time-reversal invariant photonic systems inspired by the physics of topological insulators. A theoretical prediction indicates that a meta-crystal composed of a certain type of bi-anisotropic cylinders holds a helical-edge state [20]. There, spin-dependent one-way light transport can be realized. These extensions enable us to control various waves in unprecedented manners.

## 4. Combined structure of surface magnon polaritons and photonic crystals

In the previous section, we assumed a photonic crystal composed of a magneto-optical rod array. The one-way waveguide is obtained via the gap opening due to nonzero *κ*, irrespective of polaritonic effects. It is purely a topological effect, because a non-dispersive 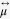 is assumed for the rods. Here, we present a combined approach using both the polaritonic effect and topological effect. With this approach, we can enlarge the band width of the one-way waveguide.

**Figure 6.**
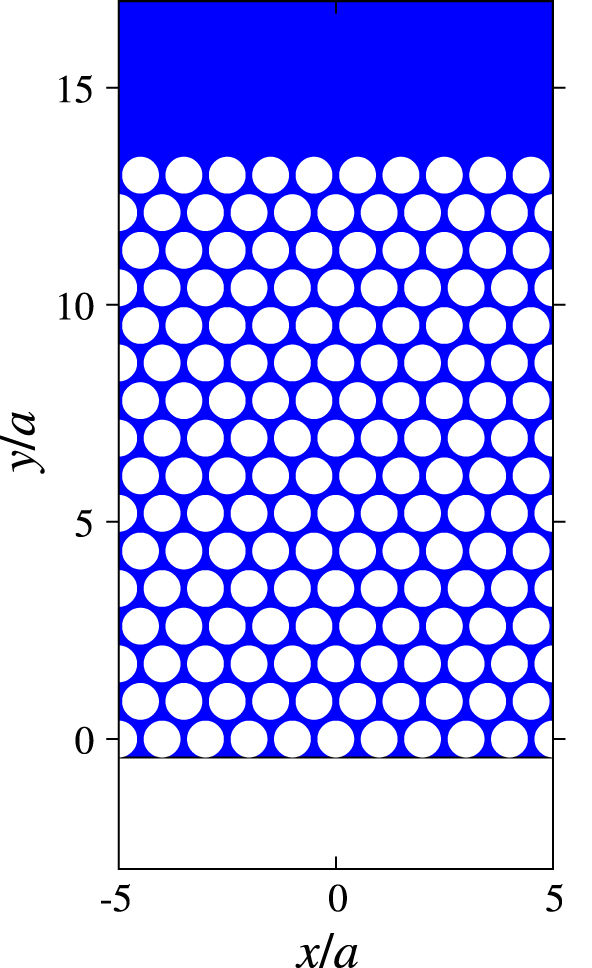
Triangular hole array in a semi-infinite ferrite material. The region outside the material is air.

The idea is as follows. Suppose we have a periodic hole array in a semi-infinite ferrite material with the flat interface at the edge as shown in figure 6. Depending on the geometry of the hole array, the photonic band structure is formed. Photonic band gaps can be found irrespective of the frequencies of the polariton gap in the ferrite material. These gaps act as new polariton gaps, in which light propagation is not allowed in the bulk. Therefore, the gaps can support ‘new’ surface magnon polaritons around the edge. The new polaritons are also affected by the topology of the photonic bands below the gap, according to the bulk-edge correspondence. Thus, the two approaches discussed in the previous sections are combined, giving us a chance to enhance the bandwidth of the one-way road for light.

**Figure 7.**
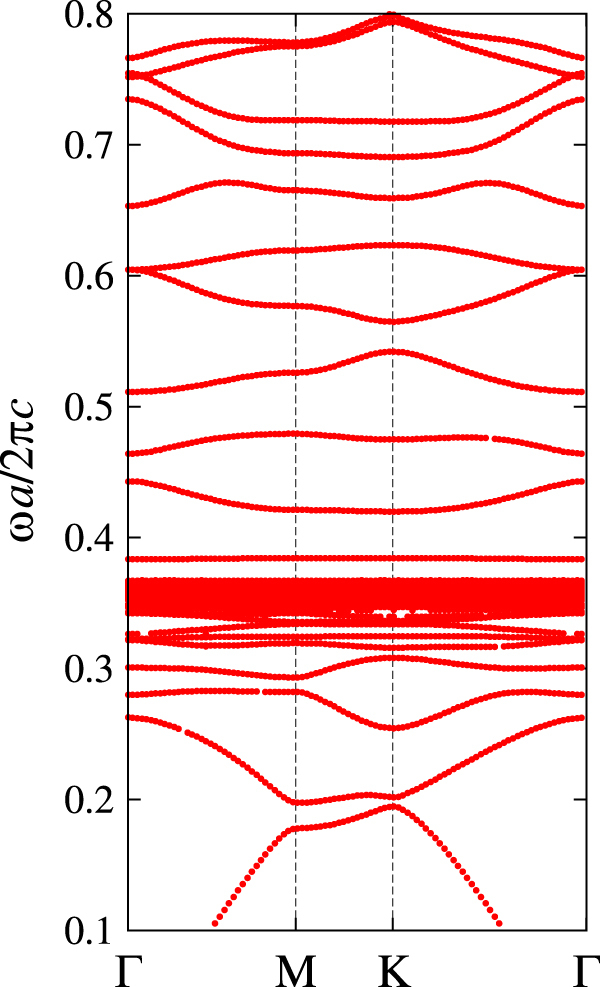
The photonic band structure of the transverse-magnetic polarization in the triangular array of circular holes with radius 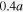 in a ferrite material. The following parameters are assumed for the ferrite material: 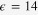, 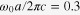 and 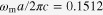. The polariton gap of the background ferrite material ranges from 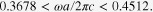

Let us consider the hole array of the triangular lattice embedded in a ferrite material. The lattice constant is chosen such that 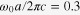 with 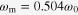. The photonic band structure of the bulk hole-array system is shown in figure 7. Since the periodic modulation by the hole array is very strong, many photonic-band gaps are formed particularly above 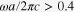. The photonic bands in this region are not necessarily topological, because the bands are separated not simply by the magneto-optical coupling. Rather, the periodic modulation in the permittivity with a large contrast seems to play a major role in the gap formation. For comparison, the polariton gap of the homogeneous ferrite material ranges from 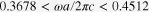. Just below the gap, a dense band region is found. This is because the refractive index 
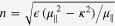
 of the ferrite becomes so large there.

**Figure 8.**
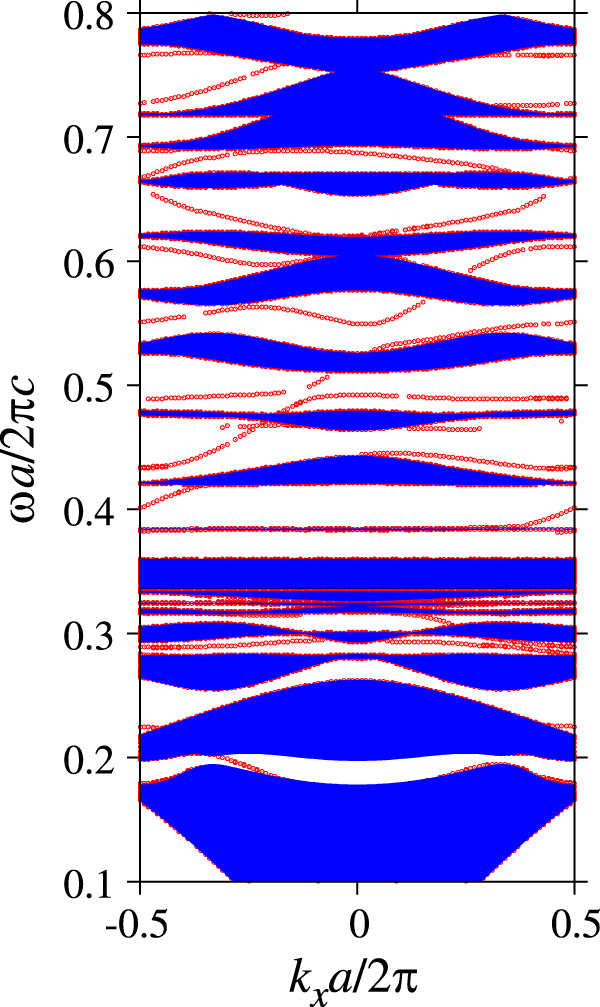
The dispersion relation of the edge states in the ferrite photonic crystal. The same parameters as in figure 7 are employed. As in figure 6, we assume a semi-infinite ferrite material with the periodic hole array of the triangular lattice, in the vicinity of the material edge. The region outside the ferrite material is air. The hole array has 16 layers along the *Γ*−M direction. The distance between the boundary hole layer and the flat interface is 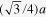. The edge states shown are localized around the flat interface. Those localized near the opposite-boundary hole layer are not observed.

The dispersion curve of the edge states is shown in figure 8. Each dot represents a peak in the optical density of states as a function of parallel momentum *k*_*x*_ to the boundary. All peaks are plotted irrespective of their peak widths. Most peaks are inside the light cone of either the air side or ferrite side. Therefore, the peaks correspond to quasi-guided edge states, which can couple with incident light. The peaks form the dispersion curves of the edge states. We can find several vacancies in the dispersion curves, because the relevant peaks become broad, or merge with the singularity of the Wood anomaly (found in the diffraction thresholds).

A striking feature is found above 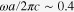. There, photonic-band gaps are relatively wide and the dispersion curves of the edge states are clearly visible. It is remarkable that the edge-state dispersion curves are almost continuous and chiral (having positive slopes) in a wide frequency range or 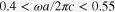, taking account of the periodicity in the surface Brillouin zone. Although the curves are separated by the photonic band regions, the band-width/mid-band ratio of the one-way band is about 0.3 if the intercepting photonic band regions are neglected. This value is much larger than that in the homogeneous ferrite material, which is just 0.02.

Therefore, the periodic hole array near the edge of the semi-infinite ferrite material supports the broad-band one-way waveguide of the modulated surface magnon polariton.

## 5. Conclusions

We have presented constructions of optical one-way waveguides using the surface polaritons and topological effect. They use the magneto-optical coupling in the permittivity or permeability tensors in common. The construction via the polariton utilizes the polaritonic gap, and the resulting band of the one-way waveguide is very limited both in its frequency range and band width. The topological construction utilizes photonic crystal, and is free from these limitations. However, the resulting one-way band depends strongly on the magneto-optical coupling of the optical substances used in the photonic crystal. We propose a combined design of these two approaches, using a periodic hole array in a semi-infinite ferrite material. It enables us to construct a broad-band one-way waveguide, in the vicinity of the edge of the semi-infinite specimen.
